# Use of Digital COVID-19 Exposure Notifications at a Large Gathering: Survey Analysis of Public Health Conference Attendees

**DOI:** 10.2196/50716

**Published:** 2024-03-18

**Authors:** Caitlin M Drover, Adam S Elder, Brandon L Guthrie, Debra Revere, Nicole L Briggs, Laura M West, Amanda Higgins, William B Lober, Bryant T Karras, Janet G Baseman

**Affiliations:** 1 Department of Epidemiology School of Public Health University of Washington Seattle, WA United States; 2 Department of Global Health School of Public Health University of Washington Seattle, WA United States; 3 Department of Health Systems and Population Health School of Public Health University of Washington Seattle, WA United States; 4 Department of Health Washington State Tumwater, WA United States; 5 Department of Biobehavioral Nursing and Health Informatics School of Nursing University of Washington Seattle, WA United States; 6 Department of Biomedical Informatics and Medical Education School of Medicine University of Washington Seattle, WA United States

**Keywords:** COVID-19, exposure notification, digital public health tool, survey analysis, conference, online survey, digital tool, public health, contact tracing

## Abstract

**Background:**

WA Notify was Washington State’s smartphone-based COVID-19 digital exposure notification (EN) tool, which was used to help limit the spread of COVID-19 between November 30, 2020, and May 11, 2023. Following the 2022 Washington State Public Health Association Annual Conference, attendees who had WA Notify activated began receiving ENs alerting them to a possible COVID-19 exposure during the conference. A survey was emailed to all conference attendees to measure WA Notify adoption, mechanisms through which attendees received ENs, and self-reported engagement in protective behaviors postexposure.

**Objective:**

This study aimed to learn more about the experiences of WA Notify adopters and nonadopters who may have been exposed to COVID-19 at a large group gathering.

**Methods:**

A web-based survey administered through REDCap (Research Electronic Data Capture; Vanderbilt University) was sent to all attendees of the Washington State Public Health Association conference. Self-reported demographic information and characteristics of respondents were summarized. Regression models were used to estimate relative risks to compare WA Notify adoption and testing behaviors between groups.

**Results:**

Of the 464 total registered attendees who were sent the survey, 205 (44%) responses were received; 201 eligible attendees were included in this analysis. Of those, 149 (74%) respondents reported having WA Notify activated on their phones at the time of the conference. Among respondents with WA Notify activated, 54% (n=77) reported learning of their potential exposure from a WA Notify EN. Respondents who reported that they did not have WA Notify activated and learned of their potential exposure via the event-wide email from conference organizers were 39% less likely to test for COVID-19 compared to respondents with WA Notify activated who learned of their potential exposure from the email (relative risk 0.61, 95% CI 0.40-0.93; *P*=.02), and this gap was even larger when compared to respondents who learned of their exposure from a WA Notify EN. The most commonly cited reason for not having WA Notify activated was privacy concerns (n=17, 35%), followed by not wanting to receive ENs (n=6, 12%) and being unaware of WA Notify (n=5, 10%).

**Conclusions:**

Digital EN systems are an important tool to directly and anonymously notify close contacts of potential exposures and provide guidance on the next steps in a timely manner. Given the privacy concerns, there is still a need for increasing transparency surrounding EN technology to increase uptake by the public if this technology were to be used in the future to slow the spread of communicable diseases.

## Introduction

### Background

Smartphone-based COVID-19 digital exposure notification (EN) tools were developed at the beginning of the COVID-19 pandemic to help limit the spread of the SARS-CoV-2 virus. These tools alert users of possible exposure to COVID-19 based on Bluetooth-based proximity to other users’ devices and other users informing the system of a positive COVID-19 diagnosis [1]. According to the Association of Public Health Laboratories, which helped support EN systems across the United States, 28 states have implemented a digital EN tool, including Washington State, which launched its EN system, WA Notify on November 30, 2020 [2,3]. Like most of these tools, WA Notify was built using the Google-Apple Exposure Notification (GAEN) Bluetooth system and its GAEN Express platform [1-4]. GAEN-based digital COVID-19 EN tools preserve the users’ privacy; adoption or activation of the tool is anonymous, users and their locations are not tracked, and data protections ensure ENs are distributed anonymously. As of April 11, 2023, WA Notify had been activated on smartphones over 3.92 million times. During the period in which WA Notify was operating, approximately 230,000 positive COVID-19 cases were reported to the system and an estimated 1.94 million ENs were shown to users [5].

The effectiveness of EN tools like WA Notify is presumed to include providing rapid notification of possible exposure to users [6] and using effective strategies to encourage individuals shown an EN to engage in behaviors to reduce further spread, such as testing and isolating [7,8]. While direct observation of WA Notify users’ protective behaviors after being shown an EN is not possible, we can ask users who were shown an EN about their anticipated and completed protective behaviors through optional, anonymous surveys. The results from these surveys suggest that ENs do influence behavior, and it has been estimated that digital EN tools such as WA Notify have averted thousands of new COVID-19 cases in the states in which they have been deployed and widely adopted [9-11].

In October 2022, the Washington State Public Health Association (WSPHA) held its Annual Conference. Several conference attendees tested positive for COVID-19 either during or following the conference. In the week following, WA Notify users who attended the conference began receiving ENs of possible exposure during the time period when they were attending the conference. This event presented a unique opportunity to capture WA Notify user interactions in the context of a large public gathering as well as the experiences of public health professionals—a population potentially more likely to both adopt digital EN tools and engage in protective behaviors—undertaking protective health behaviors in response to receiving an EN. We describe the results of a postconference web-based survey emailed to all attendees to measure WA Notify adoption, mechanisms through which attendees were alerted to a possible COVID-19 exposure (digital EN, personal communication, etc), and self-report of engagement in protective behaviors after learning of the exposure.

### Objectives

We aimed to learn more about the experiences of WA Notify adopters and nonadopters who may have been exposed to COVID-19 at a large group gathering.

## Methods

### How WA Notify Works

WA Notify (and other similar EN systems) can be activated either through the settings on iPhones or downloaded as an app on Android phones [3,5]. When in close proximity to another activated phone, the devices will exchange random, anonymous cryptographic Bluetooth keys. If the owner of either device later tests positive for COVID-19, they may anonymously confirm their positive result using the tool. Depending on the length and proximity of the phones’ interaction, ENs will be shown on phones belonging to individuals who have exchanged Bluetooth keys with the infected individual. While ENs may be displayed on phones for exposures happening up to 10 days earlier, the average time between exposure and the EN alert was 4.6 days in October 2022. The examples of EN messages from WA Notify can be found in Multimedia Appendix 1.

### Survey Instrument

A web-based survey was designed to capture WA Notify engagement (adoption and reasons for not having WA Notify activated), awareness that someone who attended the conference later tested positive for COVID-19, respondents’ COVID-19 status (presence of symptoms, testing, and result), vaccination status, and demographics (race or ethnicity, age, gender, and state residency). The survey was piloted internally to assess clarity, question order, and timing. The final survey was programmed and administered through REDCap (Research Electronic Data Capture) [12]. A copy of the survey can be found in Multimedia Appendix 2.

Survey invitations were distributed via email to a list of all WSPHA conference attendees on October 28, 2022, a total of 15 days after the conference, by the conference organizer. Reminders were sent on November 6, 2022, and the survey closed on November 10, 2022.

### Inclusion Criteria

Only respondents who attended the WSPHA Annual Conference on October 11 through 13, 2022, were included. Reports of ENs distributed outside of October 11 through 23 were excluded since these ENs fall outside of the 10-day window for ENs plausibly resulting from interactions at the WSPHA conference [3].

### Statistical Analysis

Survey responses were tallied and stratified by the primary outcome of interest, WA Notify activation status. Variables of interest included how respondents learned that someone who attended the conference later tested positive for COVID-19, the development of COVID-19 symptoms, engagement in protective behaviors after being shown an EN, and vaccination status. Univariate Poisson regression models were used to estimate relative risks (RRs) of having WA Notify activated and postconference testing behavior between groups.

### Ethical Considerations

The University of Washington institutional review board determined that this project was a public health quality improvement or surveillance project and nonhuman subjects research. The data used in this study were obtained from a voluntary survey that did not provide any compensation for participation and did not collect any personally identifiable information.

## Results

### Respondent Demographics and Characteristics

#### Demographics

Of the 464 total registered attendees who were sent the survey, 205 (44%) responses were received. Four surveys were excluded because the respondents did not attend the conference in person. A total of 201 completed surveys were included in the analysis, of which 149 (74%) reported having WA Notify active on their phones during the conference (Table 1).

The majority of the sample identified as female (n=158, 79%), White (n=156, 78%), and non-Hispanic, any race (n=174, 87%). Sixty-four percent (n=128) resided in Western Washington and 92% (n=184) reported having completed the primary COVID-19 vaccination series and receiving at least 1 booster.

**Table 1:** Respondent characteristics by WA Notify activation status (N=201).

		WA Notify activated		
		Yes (n=149)	No (n=49)	Total^a^ (N=201)
**Age (years), n (%)**				
	18-34	55 (36.9)	9 (18.4)	66 (32.8)
	35-44	35 (23.5)	17 (34.7)	52 (25.9)
	45-54	30 (20.1)	9 (18.4)	39 (19.4)
	55-64	26 (17.4)	8 (16.3)	34 (16.9)
	65+	3 (2.0)	5 (10.2)	9 (4.5)
	Missing	0 (0)	1 (2)	1 (0.5)
**Gender, n (%)**				
	Male	23 (15.4)	9 (18.4)	33 (16.4)
	Female	119 (79.9)	37 (75.5)	158 (78.6)
	Nonbinary	3 (2)	1 (2)	4 (2)
	Prefer not to answer	3 (2)	0 (0)	3 (1.5)
	Missing	1 (0.7)	2 (4.1)	3 (1.5)
**Race^b^, n (%)**				
	American Indian or Alaska Native	4 (2.7)	2 (4.1)	6 (3)
	Asian	18 (12.1)	1 (2)	19 (9.5)
	Black	9 (6)	3 (6.1)	12 (6)
	Native Hawaiian or Pacific Islander	0 (0)	0 (0)	0 (0)
	White	113 (75.8)	41 (83.7)	156 (77.6)
	Other	8 (5.4)	0 (0)	9 (4.5)
	Missing	4 (2.7)	4 (8.2)	8 (4)
**Hispanic ethnicity^c^, n (%)**				
	Hispanic	16 (10.7)	4 (8.2)	21 (10.4)
	Not Hispanic	130 (87.2)	42 (85.7)	174 (86.6)
	Missing	3 (2)	3 (6.1)	6 (3)
**Region, n (%)**				
	Eastern Washington	26 (17.4)	15 (30.6)	41 (20.4)
	Western Washington	103 (69.1)	24 (49)	128 (63.7)
	Missing	20 (13.4)	10 (20.4)	32 (15.9)
**Work from home status, n (%)**				
	All of the time	61 (40.9)	21 (42.9)	84 (41.8)
	Most of the time	42 (28.2)	7 (14.3)	50 (24.9)
	Some of the time	34 (22.8)	14 (28.6)	48 (23.9)
	Not at all	12 (8.1)	7 (14.3)	19 (9.5)
**Vaccination status, n (%)**				
	Unvaccinated	2 (1.3)	0 (0)	2 (1)
	Primary series only	6 (4)	8 (16.3)	15 (7.5)
	Primary series+booster	141 (94.6)	41 (83.7)	184 (91.5)
**Days since the last dose**				
	Mean (SD)	124 (152)	154 (191)	134 (165)
	Median (IQR)	42.0 (0-665)	48.5 (0-772)	43.5 (0-772)
	Missing, n (%)	6 (4)	3 (6.1)	9 (4.5)

^a^Three respondents reported “I’m not sure” to whether they had WA Notify activated on their phones.

^b^Respondents could select more than 1 answer and percentages can add to over 100%.

^c^Hispanic origin is considered here as a grouping often considered by public health researchers and the United States Census Bureau.

#### WA Notify Adoption

Reported WA Notify adoption was highest among younger age groups, with 86% (n=55) of those 18-34 years old having WA Notify activated on their smartphones compared to 38% (n=3) of those 65 years and older (Table 2). We found that those aged 35-44 years were 22% less likely to have WA Notify activated than those aged 18-34 years (RR 0.78, 95% CI 0.63-0.97; *P=*.03).

Reported adoption also varied across racial groups. Respondents who identified as Asian were 29% more likely to have WA Notify activated compared to those who did not identify as Asian (RR 1.29, 95% CI 1.13-1.49; *P<*.001). Respondents who identified as “other” race were 35% more likely to have WA Notify activated compared to those who did not identify as “other” (RR 1.35, 95% CI 1.24-1.47; *P<*.001). We found no statistically significant association between WA Notify adoption and Hispanic ethnicity of any race.

Respondents living in Eastern Washington were 22% less likely to have WA Notify activated compared to those living in Western Washington (RR 0.78, 95% CI 0.61-1.00; *P*=.05).

**Table 2:** Respondent characteristics associated with having WA Notify activated (n=198).

Characteristics		Values, n/N (%)		Risk ratio (95% CI)		*P* value
**Age (years)**						
	18-34	55/64 (86)	1 (Reference)		Reference	
	35-44	35/52 (67)	0.78 (0.63-0.97)		*.03^a^*	
	45-54	30/39 (77)	0.90 (0.73-1.09)		.27	
	55-64	26/34 (77)	0.89 (0.72-1.10)		.28	
	65+	3/8 (38)	0.44 (0.18-1.07)		.07	
**Gender**						
	Male	23/32 (72)	1 (Reference)		Reference	
	Female	119/156 (76)	1.06 (0.84-1.34)		.62	
	Nonbinary	3/4 (75)	1.04 (0.57-1.91)		.89	
**Race^b^**						
	American Indian or Alaska Native	4/6 (67)	0.88 (0.50-1.56)		.67	
	Asian	18/19 (95)	1.29 (1.13-1.49)		*<.001*	
	Black	9/12 (75)	1.00 (0.71-1.40)		.98	
	White	113/154 (73)	0.90 (0.76-1.06)		.21	
	Other	8/8 (100)	1.35 (1.24-1.47)		*<.001*	
**Hispanic ethnicity**						
	Not Hispanic	130/172 (76)	1 (Reference)		Reference	
	Hispanic	16/20 (80)	1.06 (0.84-1.34)		.64	
**Region**						
	Western Washington	103/127 (81)	1 (Reference)		Reference	
	Eastern Washington	26/41 (63)	0.78 (0.61-1.00)		.05	
**Work from home status**						
	All of the time	61/82 (74)	1.18 (0.82-1.70)		.38	
	Most of the time	42/49 (86)	1.36 (0.95-1.95)		.10	
	Some of the time	34/48 (71)	1.12 (0.76-1.65)		.56	
	Not at all	12/19 (63)	1 (Reference)		Reference	
**Vaccination status**						
	Unvaccinated	2/2 (100)	N/A^c^		N/A	
	Primary series only	6/14 (43)	0.55 (0.30-1.02)		.06	
	Primary series+booster	141/182 (78)	1 (Reference)		Reference	

^a^Italic formatting in this table indicates that the β coefficient from the corresponding regression is significantly different from 0 at the *P*=.05 level.

^b^Respondents could select more than 1 answer. The reference category for each comparison was those who did not select each respective response.

^c^N/A: not available.

#### COVID-19 Vaccination Status

Only 2 respondents reported being unvaccinated (Table 2). Respondents who reported receiving only the primary vaccination series were 45% less likely to have WA Notify activated than those with at least 1 booster, although this association was not statistically significant (RR 0.55, 95% CI 0.30-1.02; *P*=.06).

#### EN Receipt

**Figure 1:**
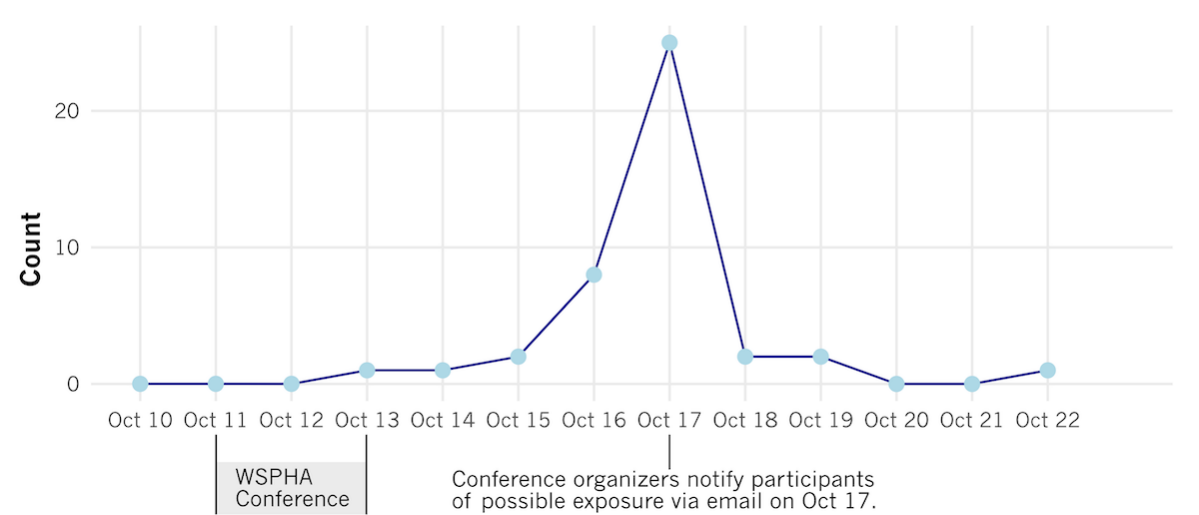
The plausible dates for an exposure notification shown because of potential exposure during the conference were between October 11 and 23 due to the 10-day storage period for keys used to show exposure notifications. Data were only available for respondents with WA Notify activated who remembered the date of the first WA Notify exposure notification received. Three respondents reported exposure notifications outside of this range (October 2, 10, and 31), and these exposure notifications were not included in the analysis. WSPHA: Washington State Public Health Association.

The WSPHA Annual Conference took place from October 11 through 13, 2022, in Wenatchee, WA. From October 13 through October 23, there were 42 conference attendees who reported receiving their first WA Notify EN. Figure 1 illustrates the dates of the first reported WA Notify EN among participants who remember the date of the EN and who had WA Notify activated during this period. We do not know when attendees tested positive or confirmed their positive tests using WA Notify. However, due to the 10-day storage period for keys used to determine exposure, the plausible dates for an EN shown due to a potential exposure during the conference were between October 11 and 23. Three respondents reported ENs outside of this range (October 2, 10, and 31), and these ENs were not included in the analysis.

The only EN dates collected with this survey were for the first WA Notify EN shown to those who reported having WA Notify activated and who reported being aware that someone who attended the conference later tested positive for COVID-19. Dates for other ENs were unavailable, except for the WSPHA conference organizer email EN sent to all attendees on October 17.

#### Symptoms, Testing, and WA Notify Adoption

Following the conference, 17% (n=35) of respondents reported experiencing at least 1 symptom and 63% (n=126) took a test for COVID-19 (Table 3).

Among those who tested, 92.1% (n=116) reported taking an “at-home” rapid antigen test for COVID-19, while the others received a polymerase chain reaction test. Testing was most frequently prompted by receiving an EN from WA Notify (n=58, 29%) or email notification from WSPHA conference organizers (n=56, 28%), although many respondents also reported testing being a routine behavior when they travel (n=44, 22%). Respondents who reported having WA Notify activated were 74% more likely to test for COVID-19 following the conference compared to those who did not have WA Notify activated (RR 1.74, 95% CI 1.22-2.47; *P*=.002). Nine (7%) respondents reported testing positive following the conference.

**Table 3:** Respondent testing behaviors and postconference health outcomes by WA Notify activation status (N=201).

		WA Notify activated, n (%)		
		Yes (n=149)	No (n=49)	Total^a^ (N=201)
**Symptoms postconference**				
	0	112 (75.2)	35 (71.4)	148 (73.6)
	1	8 (5.4)	3 (6.1)	11 (5.5)
	2+	19 (12.8)	4 (8.2)	24 (11.9)
	Missing	10 (6.7)	7 (14.3)	18 (9)
**Tested postconference**				
	Yes	105 (70.5)	20 (40.8)	126 (62.7)
	No	43 (28.9)	29 (59.2)	74 (36.8)
	Missing	1 (0.7)	0 (0)	1 (0.5)
**Test reason^b,c^**				
	WA Notify EN^d^	56 (37.6)	2 (4.1)	58 (28.9)
	Conference EN	44 (29.5)	12 (24.5)	56 (27.9)
	Personal EN	14 (9.4)	1 (2)	15 (7.5)
	Symptoms	13 (8.7)	3 (6.1)	16 (8)
	Routine	38 (25.5)	6 (12.2)	44 (21.9)
	Other	11 (7.4)	2 (4.1)	14 (7)
**Test method^c^**				
	Home rapid test	96 (91.4)	19 (95)	116 (92.1)
	Testing center	9 (8.6)	1 (5)	10 (7.9)
**Tested positive^c^**				
	Yes	7 (6.7)	1 (5)	9 (7.1)
	No	98 (93.3)	19 (95)	117 (92.9)

^a^Three respondents reported “I’m not sure” to whether they had WA Notify activated on their phones.

^b^Respondents could select more than 1 answer and percentages can add to over 100%.

^c^Among respondents who reported testing for COVID-19 following the conference (n=126).

^d^EN: exposure notification.

#### Pathways for Receiving First EN

Most respondents (n=194, 97%) reported that they were aware that someone who attended the conference later tested positive for COVID-19 (Table 4).

Respondents learned about the attendees who tested positive for COVID-19 through a variety of pathways. Among those who were aware, respondents most frequently reported learning of the COVID-19–positive attendees via an event-wide email notification from WSPHA conference organizers (n=93, 48%) or receiving a WA Notify EN (n=77, 40%). A small number of respondents learned about the COVID-19–positive attendees via personal communication from another attendee who tested positive for COVID-19 (n=7, 4%), communication from someone else who had been exposed (n=6, 3%), or an internal email notification from the Washington State Department of Health (n=4, 2%). Among respondents with WA Notify activated, 54% (n=77) reported learning of their exposure from WA Notify, while 33% (n=48) learned of their exposure from the conference organizer email. It is possible that some portion of the 33% (n=48) of respondents with WA Notify activated who received the email notification first were not considered exposed based on Bluetooth proximity or the risk score used in WA Notify. Alternatively, this group may have also been shown a WA Notify EN after the email notification, but data were not collected on additional notifications.

**Table 4:** Awareness of conference attendees who later tested positive for COVID-19 by WA Notify activation status (N=201).

		WA Notify activated, n (%)		
		Yes (n=149)	No (n=49)	Total^a^ (N=201)
**Aware of COVID-19–positive attendees**				
	Yes	144 (96.6)	47 (95.9)	194 (96.5)
	No	5 (3.4)	1 (2)	6 (3)
	Missing	0 (0)	1 (2)	1 (0.5)
**How COVID-19–positive attendees first heard about^b^**				
	Email from WSPHA^c^ conference organizers	48 (33.3)	42 (91.3)	93 (48.2)
	WA Notify EN^d^	77 (53.5)	0 (0)	77 (39.9)
	Personal communication from a COVID-19–positive attendee	7 (4.9)	0 (0)	7 (3.6)
	Notified by a contact tracer	0 (0)	0 (0)	0 (0)
	Other^e^	12 (8.3)	4 (8.7)	16 (8.3)

^a^Three respondents reported “I’m not sure” to whether they had WA Notify activated on their phones.

^b^Among respondents who were aware that someone who attended the conference later tested positive for COVID-19 (n=194).

^c^WSPHA: Washington State Public Health Association.

^d^EN: exposure notification.

^e^Reasons included communication from someone else who was exposed (n=6), Washington State Department of Health email notification (n=4), and cannot remember (n=2).

### Associations Among Pathway of First Received EN, Symptoms, Vaccination History, and Testing

Respondents who reported that they did not have WA Notify activated and learned that someone who attended the conference later tested positive for COVID-19 via the email from conference organizers were 39% less likely to take a test for COVID-19 than respondents with WA Notify activated who learned of their potential exposure from the email (RR 0.61, 95% CI 0.40-0.93; *P*=.02; Table 5).

Respondents with WA Notify activated who learned of their potential exposure from a WA Notify EN were slightly more likely to test compared to those with WA Notify who received the email notification first, although this association was not significant (RR 1.14, 95% CI 0.90-1.45; *P*=.28).

The number of symptoms experienced was associated with testing for COVID-19 following the conference. Respondents who reported experiencing 1 symptom were 62% more likely to test than those who experienced no symptoms (RR 1.62, 95% CI 1.28-2.05; *P*<.001), and those who experienced 2 or more symptoms were 78% more likely to test than those who experienced no symptoms (RR 1.78, 95% CI 1.55-2.06; *P*<.001; Table 5). With the exception of 1 person who reported a sore throat, everyone who reported symptoms also reported testing for COVID-19.

**Table 5:** Associations among the method of receiving an EN^a^, symptoms, vaccination history, and testing postconference (N=201).

Characteristic		Postconference testing, n/N (%)	RR^b^ (95% CI)	*P* value
**How COVID-19–positive attendees first heard about^c^**				
	WA Notify EN	58/77 (75)	1.14 (0.90-1.45)	.28
	Email from WSPHA^d^ conference organizers—WA Notify activated	31/47 (66)	1 (Reference)	Reference
	Email from WSPHA conference organizers—WA Notify not activated	17/42 (41)	0.61 (0.40-0.93)	*.02^e^*
**Symptoms**				
	0	83/148 (56)	1 (Reference)	Reference
	1	10/11 (91)	1.62 (1.28-2.05)	*<.001*
	2+	23/23 (100)	1.78 (1.55-2.06)	*<.001*
**Vaccination status**				
	Unvaccinated	0/2 (0)	N/A^f^	N/A
	Primary series only	7/15 (47)	0.72 (0.41-1.25)	.24
	Primary series+booster	119/183 (65)	1 (Reference)	Reference
**Days since last the vaccine dose**				
	30-day difference	N/A	0.98 (0.96-1.01)	.13

^a^EN: exposure notification.

^b^RR: relative risk.

^c^Among respondents who were aware that someone who attended the conference later tested positive for COVID-19 (n=194).

^d^WSPHA: Washington State Public Health Association.

^e^Italic formatting in this table indicates that the β coefficient from the corresponding regression is significantly different from 0 at the *P*=.05 level.

^f^N/A: not available.

### Associations Between Pathway of First Received EN and Developing COVID-19

No statistically significant association was found between how respondents learned of the conference attendees who later tested positive for COVID-19 and whether the respondent developed COVID-19 using the Fisher exact test (*P*=.21; Table 6).

**Table 6:** Associations between the method of receiving an EN^a^ and developing COVID-19 among those who were aware of COVID-19–positive attendees and tested following the conference (N=120).

Characteristic		Values, n/N (%)	*P* value^b^	
**How COVID-19–positive attendees first heard about^c^**				.21
	Email from WSPHA^d^ conference organizers	6/49 (12)		
	WA Notify EN	2/58 (3)		
	Personal communication from a COVID-19–positive attendee	1/6 (17)		
	Other	0/7 (0)		

^a^EN: exposure notification.

^b^Fisher exact *P* value.

^c^There was an option for “Notified by a contact tracer,” but 0 respondents selected it.

^d^WSPHA: Washington State Public Health Association.

### Reasons for Not Activating WA Notify

Among those who reported not having WA Notify activated (N=49), there were a variety of reasons reported with some respondents (n=7) reporting multiple reasons. The most commonly cited reason was privacy concerns (n=17, 35%), followed by not wanting to receive ENs (n=6, 12%), and being unaware of WA Notify (n=5, 10%). Other reasons for not activating WA Notify included not understanding it (n=3, 6%), thinking it is cumbersome or bothersome (n=3, 6%), not getting around to it (n=3, 6%), having limited phone storage (n=3, 6%), not living in WA State, limited contact with others (n=2, 4%), and assumed exposure (n=2, 4%).

## Discussion

### Overview

This analysis allowed us to compare the characteristics of WA Notify adopters and nonadopters among those attending the WSPHA Annual Conference. It also explored testing behavior following a possible exposure and factors that may influence EN tool uptake. Our survey population consisted of public health professionals who, compared to the general public, are likely to be more aware of COVID-19 risks and prevention measures and are more inclined to use digital EN tools.

### Principal Results

Our findings indicate that respondents who identified as WA Notify users were more likely to be younger compared to their counterparts, although this is unsurprising as younger age groups may be more comfortable with digital tools and technology in general. WA Notify users were also more likely to receive COVID-19 vaccinations relative to their counterparts, which would align with a higher likelihood of taking health precautions in general. Key findings included high levels of reported privacy concerns among nonadopters, that the majority (n=77, 54%) of WA Notify users reported first learning about their possible exposure from the tool, and that WA Notify users were more likely to test for COVID-19 compared to nonusers regardless of how they were first notified about their exposure.

Among nonusers, the most common reason for not having WA Notify activated was privacy concerns (n=17, 35%), despite stringent privacy protections, which make it nearly impossible for any government agency or corporation to obtain individual-level information [13,14]. We recognize that social desirability bias may have led nonusers to more frequently cite privacy concerns for not adopting WA Notify and assume that the general public, whose affiliation with public health is lower, likely has equal or greater privacy concerns than our sample. Still, this finding underscores the need for improved communications surrounding the privacy and data security of digital EN systems.

WA Notify users were significantly more likely to test for COVID-19 irrespective of how they first learned of their potential exposure. It is possible that testing was encouraged by the more personalized WA Notify EN or receiving multiple notifications or the combination of these 2 factors. Unfortunately, we cannot determine the extent to which WA Notify ENs encouraged testing. It is possible individuals with WA Notify activated were also more likely to test for COVID-19 regardless of whether they received an EN.

However, our findings indicate that WA Notify played an important role in distributing timely ENs to conference attendees following COVID-19 exposures at the WSPHA conference. Additionally, we believe a proximity-based digital EN system could be particularly useful in other large gatherings to alert close contacts of their exposure and reduce further transmission. The WSPHA conference was unique in that the attendees were public health professionals, and the sponsoring organization’s mission focused on health and public safety. Organizers in other event contexts may not be informed about infectious individuals attending the event, and if informed, organizers may be unable to or choose not to distribute their own EN to attendees.

Our results add to the evidence of the value of digital EN systems, but work remains to encourage adoption and engagement with these systems. The adoption of these systems is influenced by how easy they are to use (which is improved by the GAEN Express platform). Additionally, conducting pilot studies of the system, strong advertising campaigns, and engaging a wide array of stakeholders all likely contributed to WA Notify’s successes and can improve the adoption of similar systems in the future [3].

### Limitations

There are several limitations to our findings. The survey population was a subset of individuals from the WSPHA conference and thus could have limited generalizability to the general population since the survey respondents are likely highly invested in public health and public health measures. These individuals could be more likely to use EN tools and take additional precautions against COVID-19 infection compared to the population of all WSPHA conference attendees or the general public.

There are also limitations to our survey design. The survey did not ask about prior COVID-19 infection, so natural immunity could not be accounted for. The question about vaccination status allowed an option for primary series+booster; respondents who selected that response may have received either 1 booster or multiple boosters because the new bivalent booster had recently become available. We did not assess the respondents’ role within public health, such as if they worked at the state or local level or were a student. Knowing these characteristics may have provided more insight into the differences between these groups. When assessing the type and number of ENs received, data were only collected regarding the first EN method. The EN date information was only available for those who reported receiving a WA Notify EN; dates of other ENs were unknown if multiple were received.

### Conclusions

Digital EN systems are important tools that have been used to notify close contacts of potential COVID-19 exposures. Our data suggest that having WA Notify activated encouraged individuals to engage in postexposure protective behaviors relative to those who did not have the tool activated. For digital EN technologies to sustain and expand their use in slowing the spread of communicable diseases moving forward, more work must be done to encourage uptake in groups less comfortable with the technology. Developing clear, consistent, and transparent messaging around the privacy and data security protocols built into these tools is needed to reduce these barriers to adoption. Additionally, understanding how to communicate evidence of public health value both to the general public and within the public health community is needed. Investment in future digital public health tools would benefit from an improved evidence base that includes systematic research on the impact of tailored messaging and the attributes and components of such messaging for encouraging the adoption and sustained use of public health digital tools like WA Notify.

## Appendix

Multimedia Appendix 1 Examples of EN messages from WA Notify.

Multimedia Appendix 2 REDCap survey sent to conference participants.

## Abbreviations

EN: exposure notification
GAEN: Google-Apple Exposure Notification
REDCap: Research Electronic Data Capture
RR: relative risk
WSPHA: Washington State Public Health Association
