# Supplementary figures and images for: Use of Digital COVID-19 Exposure Notifications at a Large Gathering: Survey Analysis of Public Health Conference Attendees
Source: JMIR Form Res. 2024 Mar 18;8:e50716. doi: 10.2196/50716 (PMC10953810; doi:10.2196/50716)

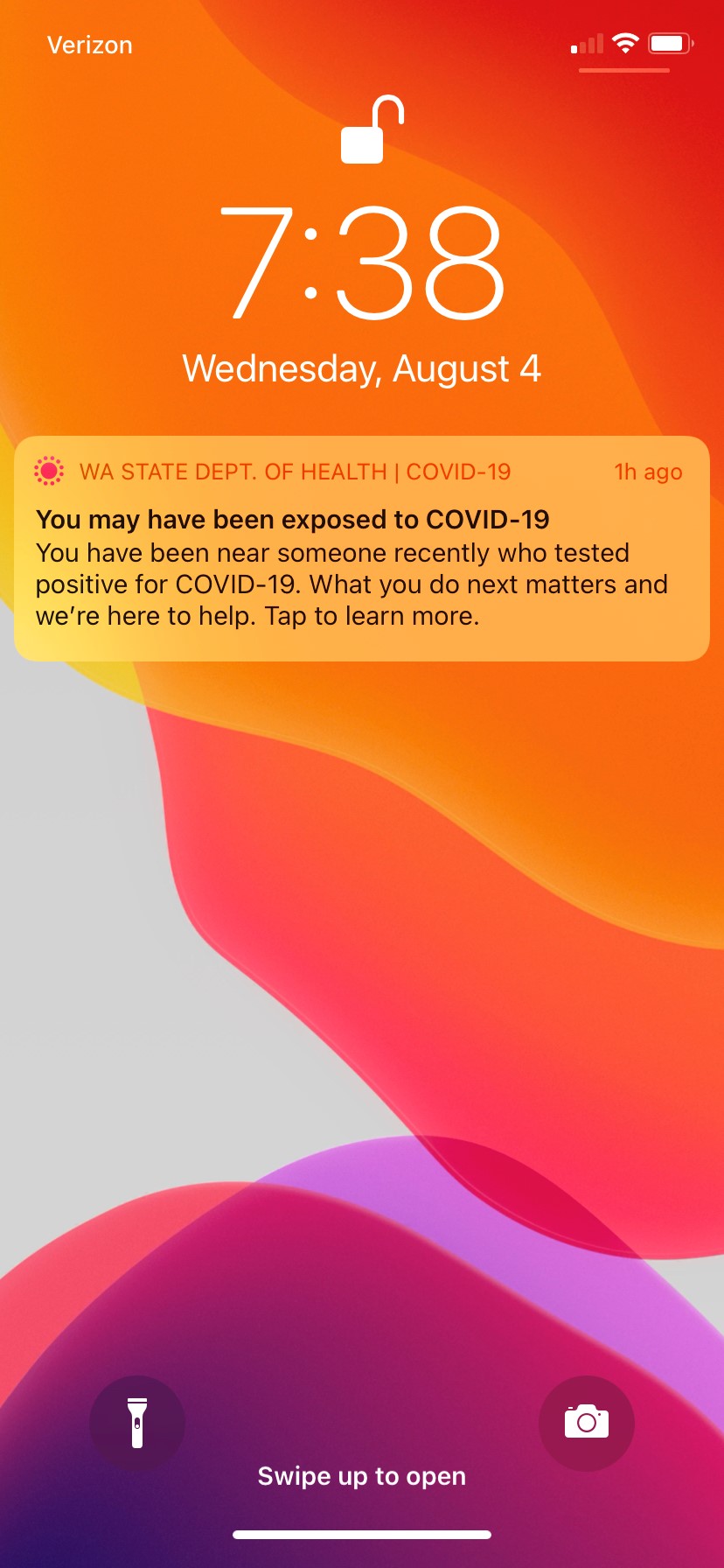

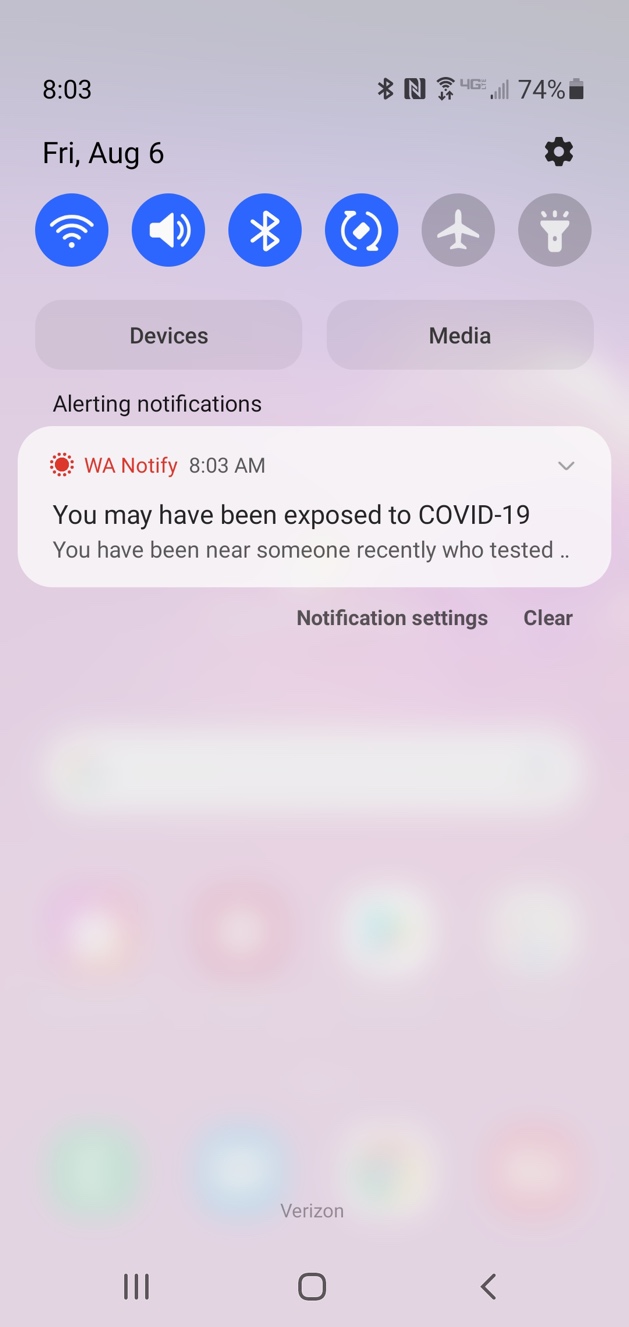

Supplement: Multimedia Appendix 1 [file formative_v8i1e50716_app1.docx]
