# Supplementary material for: Use of Digital COVID-19 Exposure Notifications at a Large Gathering: Survey Analysis of Public Health Conference Attendees
Source: JMIR Form Res. 2024 Mar 18;8:e50716. doi: 10.2196/50716 (PMC10953810; doi:10.2196/50716)
Supplement: Multimedia Appendix 2 [file formative_v8i1e50716_app2.pdf]

WSPHA WA Notify Survey

Thank you for participating in this survey.

We are interested in hearing from attendees at the WSPHA conference who may have received a Covid 19 exposure notification from the WA Notify system. This survey should take about 5 minutes to complete.

This survey is anonymous. Your IP address will not be collected. No one will be able to identify you or your individual responses, and no one will know whether or not you participated in the survey. Should data from this survey be published, no individually identifiable information will be disclosed.

Did you attend the WSPHA conference in Wenatchee in person at any time between October 10th and October 13th?	◯ Yes<br>◯ No
Do you have WA Notify active on your phone?<br><br>To check:<br>On ios: Settings >Exposure Notifications > Exposure Logging Status will say active<br>On Android: Open WA Notify app > App is active	◯ Yes<br>◯ No<br>◯ I'm not sure
We'd like to hear why you do you not have WA Notify on your phone. Please select all the reasons that apply.	☐ I have not heard of WA Notify<br>☐ I do not understand how WA Notify works<br>☐ I have data privacy concerns<br>☐ I do not want to be notified if I am exposed to Covid 19<br>☐ Other ______
Are you aware that a person attending the conference later tested positive for Covid 19?	◯ Yes<br>◯ No
How did you first hear about the positive Covid 19 case?	◯ Email from WSPHA conference organizers<br>◯ WA Notify exposure notification alert<br>◯ Personal communication from a Covid 19+ attendee<br>◯ Notification from a contact tracer<br>◯ Other ______
Did you receive a WA Notify exposure notification alert (regarding an exposure from October 10th - 13th)?	◯ No<br>◯ Yes (1 exposure notification)<br>◯ Yes (2 or more exposure notifications)<br>◯ I'm not sure
Do you recall when you received the first WA Notify alert?	◯ Yes<br>◯ I received a notification, but I don't recall when
When did you receive the WA Notify alert? (If you received more than one alert, please share the approximate date of the first alert you received.)	__________________________________
Did you take a Covid 19 test anytime between October 13th - October 23rd?	◯ Yes<br>◯ No

What prompted you to get tested for Covid 19 after the WSPHA conference? (check all that apply)	☐ I received an exposure notification from WA Notify<br>☐ I received an exposure notification email from the WSPHA conference<br>☐ I routinely test when I travel<br>☐ I had symptoms of Covid 19<br>☐ I heard from personal communication that someone had tested positive for Covid 19 at the WSPHA conference<br>☐ Other ______
What type of test did you use? (check all that apply)	☐ Tested at home with a rapid test<br>☐ Tested at home with a sample sent by mail to a testing center<br>☐ Tested at a COVID-19 testing center or health care facility (either PCR or rapid test)
Were any tests positive for Covid 19?	◯ Yes<br>◯ No
On which day did you test positive?<br><br>To learn how to anonymously notify others about a positive Covid 19 test, go to: https://doh.wa.gov/emergencies/covid-19/wa-notify#1	______________________________
Have you developed any of the following symptoms since October 12th? (check all that apply)	☐ I am not experiencing any of these symptoms<br>☐ Fever or chills<br>☐ Cough<br>☐ Shortness of breath or difficulty breathing<br>☐ Fatigue<br>☐ Muscle or body aches<br>☐ Headache<br>☐ New loss of taste or smell<br>☐ Sore throat<br>☐ Congestion or runny nose<br>☐ Nausea or vomiting<br>☐ Diarrhea
Have you received any Covid 19 vaccination?	◯ No<br>◯ Yes, primary series only<br>◯ Yes, primary series+booster
What is the date of your last Covid 19 vaccination?	______________________________
What is your age?	◯ 18-24 years<br>◯ 25-34 years<br>◯ 35-44 years<br>◯ 45-54 years<br>◯ 55-64 years<br>◯ 65-74 years<br>◯ 75 years and older
Do you identify as Hispanic/Latinx?	◯ Yes<br>◯ No

What race do you identify as? (check all that apply)	☐ White<br>☐ Black/African American<br>☐ Asian<br>☐ American Indian/Alaska Native<br>☐ Native Hawaiian/Pacific Islander<br>☐ Other
What gender do you identify as?	◯ Male<br>◯ Female<br>◯ Nonbinary<br>◯ Prefer not to answer
Do you currently live in Washington State?	◯ Yes<br>◯ No
What county do you live in?	◯ Asotin<br>◯ Benton<br>◯ Chelan<br>◯ Clallam<br>◯ Clark<br>◯ Columbia<br>◯ Cowlitz<br>◯ Douglas<br>◯ Ferry<br>◯ Franklin<br>◯ Garfield<br>◯ Grant<br>◯ Grays Harbor<br>◯ Island<br>◯ Jefferson<br>◯ King<br>◯ Kitsap<br>◯ Kittitas<br>◯ Klickitat<br>◯ Lewis<br>◯ Lincoln<br>◯ Mason<br>◯ Okanogan<br>◯ Pacific<br>◯ Pend Oreille<br>◯ Pierce<br>◯ San Juan<br>◯ Skagit<br>◯ Skamania<br>◯ Snohomish<br>◯ Spokane<br>◯ Stevens<br>◯ Thurston<br>◯ Wahkiakum<br>◯ Walla Walla<br>◯ Whatcom<br>◯ Whitman<br>◯ Yakima
Are you currently working from home?	◯ Yes, all of the time<br>◯ Yes, most of the time<br>◯ Yes, some of the time<br>◯ Not at all

Is there anything else you would like to say about receiving a WA Notify alert or message? Please use this space for additional comments.	__________________________________
Thank you for completing the survey. Are you willing to participate in any follow-up surveys or interviews related to this event?	◯ Yes<br>◯ No
Please enter your email address here. Your email will only be used for this purpose and will not be shared with any other individual or agency. We value your participation.	__________________________________
